# An Unusual Presentation of Hairy Cell Leukemia

**DOI:** 10.4274/tjh.galenos.2019.2018.0304

**Published:** 2019-08-02

**Authors:** Smeeta Gajendra, Bhawna Jha, Sarita Prasad, Pratibha Dhiman, Manorama Bhargava

**Affiliations:** 1 Medanta - The Medicity, Departments of Pathology and Laboratory Medicine, Gurgaon, India; 2 Medanta - The Medicity, Department of Medical Oncology and Hematology, Gurgaon, India

**Keywords:** Hairy cell leukemia, Immunophenotyping, Splenomegaly, Cytopenias

## To the Editor,

**Figure 1:**
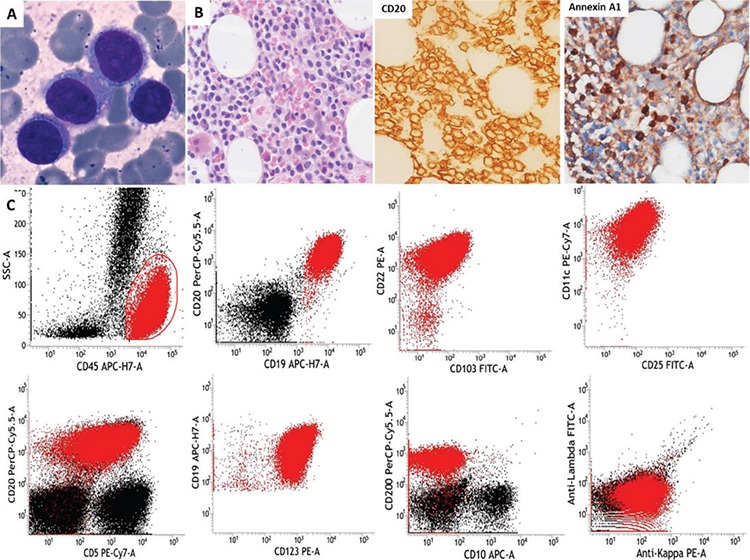
Bone marrow aspirate showing hairy cells (A: arrow). Bone marrow biopsy (B) showing abnormal lymphoid cell infiltration positive for CD20 and annexin A1. (C) Immunophenotyping showing bright CD45 positivity and further gated CD19-positive abnormal lymphoid cells, which were positive for CD20, CD22, CD103, CD11c, CD25, CD5, CD123, CD200, and kappa and negative for CD10.

The aberrant expression of CD5 in both hairy cell leukemia (HCL) and HCL-variant (HCL-v) is very rare; only 26 such cases have been reported in the literature [1]. Simultaneous absence of splenomegaly and cytopenia(s) is even rarer, which may pose a diagnostic dilemma. We describe a case of CD5-positive HCL with absence of splenomegaly and cytopenia. To the best of our knowledge, only one case of HCL without cytopenia and splenomegaly has been reported in the literature to date [2], but without CD5 positivity. Our patient was a 59-year-male, who presented with intermittent cough with expectoration for the last 3 to 4 years with no history of fever. Radiological investigations including X-ray and computed tomography scans were normal. Complete blood counts showed hemoglobin of 15.1 g/dL, white blood cell count of 7.73x109/L (neutrophils: 52%, lymphocytes: 45%, monocytes: 2%), and platelet count of 153x109/L. Peripheral blood smear (PBS) and bone marrow aspirate (BMA) showed 10% and 24% abnormal lymphoid cells, respectively (Figure 1A). These cells were small to medium in size, with abundant pale blue cytoplasm and circumferential hairy projections. Bone marrow biopsy showed interstitial aggregates of abnormal lymphoid cells (Figure 1B), which were positive for CD20 and annexin 1. Flow cytometric immunophenotyping (Figure 1C) revealed these cells to be positive for CD19, CD20, CD22, CD103, CD11c, CD123, CD25, CD5 (heterogeneous), CD200, CD23 (dim), and kappa and negative for CD10 and FMC7. The patient was found to be positive for BRAF V600E mutation. A diagnosis of HCL with aberrant CD5 was made.

HCL is an indolent small mature B lymphoid malignancy accounting for 2% of lymphoid leukemias [3]. The three most important findings for diagnosis are splenomegaly, cytopenia(s), and bone marrow dry tap resulting from marrow fibrosis [4]. In unsuspected cases with unusual presentation, the best approach for diagnosis is the careful examination of morphological details on PBS and BMA to identify the morphological features of hairy cells, which are further confirmed upon characteristic immunophenotypic profiles, as in our case. Differential diagnoses of HCL include chronic lymphocytic leukemia, prolymphocytic leukemia, splenic marginal zone lymphoma, HCL-v, and mantle cell lymphoma, which can be excluded based on characteristic morphological and immunophenotypic features. Hairy cells are 10-15 µm in diameter, with central or eccentric round, oval, or indented nuclei; reticular or netlike chromatin pattern; indistinct or absent nucleoli; pale blue cytoplasm with fine, hair-like projections or ruffled borders; and positive staining for tartrate-resistant acid phosphatase [5]. A typical combination of immunophenotypic markers expressed by hairy cells such as CD19, CD22, and CD79b, with brighter expression of CD20, along with co-expression of CD103, CD123, CD25, and CD11c, confirms the diagnosis [6].

In conclusion, this case posed a diagnostic challenge as the patient had no cytopenias or splenomegaly along with CD5 positivity. This case is important because it creates awareness of this uncommon presentation of HCL and emphasizes that the best approach in diagnosing HCL is to give careful attention to morphological details while interpreting peripheral blood, as in our case, which can prompt detailed evaluation of bone marrow with immunophenotyping in such cases for early diagnosis and management of the patient.
